# Ursodeoxycholic acid as a potential cardioprotective agent: molecular mechanisms, experimental evidence, and clinical perspectives

**DOI:** 10.3389/fphar.2026.1901120

**Published:** 2026-08-20

**Authors:** Tatjana Milivojac, Nataša Stojaković, Momir Mikov, Milorad Vujnić, Ranko Škrbić

**Affiliations:** 1 Centre for Biomedical Research, Faculty of Medicine, University of Banja Luka, Banja Luka, Bosnia and Herzegovina; 2 Department of Pathophysiology, Faculty of Medicine, University of Banja Luka, Banja Luka, Bosnia and Herzegovina; 3 Department of Pharmacology, Toxicology and Clinical Pharmacology, Faculty of Medicine, University of Banja Luka, Banja Luka, Bosnia and Herzegovina; 4 Department of Pharmacology, Toxicology and Clinical Pharmacology, Faculty of Medicine, University of Novi Sad, Novi Sad, Serbia; 5 Academy of Science and Arts of the Republic of Srpska, Department of Medical Science, Banja Luka, Bosnia and Herzegovina; 6 Department of Pathologic Physiology, First Moscow State Medical University, Moscow, Russia

**Keywords:** cardiovascular protection, endothelial dysfunction, inflammation, oxidative stress, ursodeoxycholic acid

## Abstract

Cardiovascular diseases remain the leading cause of morbidity and mortality worldwide, despite significant advances in diagnostics and pharmacotherapy. This persistent burden has shifted attention toward adjunct therapeutic strategies targeting key mechanisms of myocardial and vascular injury, including oxidative stress, mitochondrial dysfunction, endoplasmic reticulum (ER) stress, apoptosis, inflammation, endothelial dysfunction, and metabolic dysregulation. A particular interest of contemporary research is focused on bile acid (BA) signaling through nuclear and membrane receptors, primarily the Farnesoid X receptor (FXR) and Takeda G protein-coupled receptor 5 (TGR5), as central regulators of metabolic, inflammatory, and vascular responses. Ursodeoxycholic acid (UDCA), a hydrophilic BA widely used to treat hepatobiliary disorders, has emerged as a potential modulator of cardiometabolic processes. UDCA exerts direct effects through low-affinity but functionally relevant activation of the TGR5 receptor, as well as indirect effects through alterations in BA pool composition, thereby influencing FXR/TGR5 signaling pathways. Experimental studies suggest that UDCA reduces oxidative stress, stabilizes mitochondrial function, alleviates ER stress, suppresses apoptosis and inflammation, and improves endothelial function and nitric oxide (NO) bioavailability. Despite promising mechanistic evidence, currently available clinical data remain limited and are largely based on small studies and surrogate biomarkers, without confirmation of benefits in terms of major cardiovascular outcomes. This review summarizes current knowledge regarding the role of UDCA in the modulation of BA receptor signaling and its potential relevance for cardiovascular protection.

## 1 Introduction

Bile acids (BA) are a complex group of molecules whose biological role extends far beyond their classical function in the digestion and absorption of lipids. Although they were previously regarded primarily as detergents necessary for fat emulsification and the absorption of fat-soluble vitamins, contemporary research clearly demonstrates that BA are important endocrine signaling molecules with broad systemic effects (Houten et al., 2006; Hylemon et al., 2009; Li and Chiang, 2024). Their central biological function is mediated through the activation of nuclear and membrane receptors, primarily Farnesoid X receptor (FXR) and Takeda G protein-coupled receptor 5 (TGR5), through which they regulate lipid and glucose metabolism, energy homeostasis, inflammation, and immune responses (Thomas et al., 2008; Fiorucci et al., 2018; Zangerolamo et al., 2025; Dawson and Karpen, 2015; Nguyen and Bouscarel, 2008; Zhang et al., 2006). Signaling through these receptors represents the fundamental mechanistic framework underlying their systemic effects.

Primary BA, such as cholic acid (CA) and chenodeoxycholic acid (CDCA), are synthesized in the liver and secreted into the intestine, where they undergo extensive microbial transformation. Bacterial enzymes, including bile salt hydrolase, 7α-dehydroxylase, and hydroxysteroid dehydrogenase, mediate deconjugation, dehydroxylation, oxidation, and epimerization of BA (Ridlon et al., 2006; Guzior and Quinn, 2021; Ridlon et al., 2023). These reactions substantially alter BA solubility, hydrophobicity, signaling potency, and tissue toxicity.

In this context, the microbiota modulates BA signaling through two opposing mechanisms. On one hand, it promotes the formation of more hydrophobic secondary BA, such as deoxycholic acid (DCA) and lithocholic acid (LCA), which may destabilize cellular membranes, promote oxidative stress, impair mitochondrial function, and activate inflammatory pathways, thereby contributing to tissue injury (Yntema et al., 2023; Collins et al., 2023; Singh et al., 2019). Such effects may be relevant in the cardiovascular system, where BA can influence endothelial function, cardiomyocyte contractility, intracellular calcium handling, and electrical stability (Perez and Briz, 2009; Williamson et al., 2001; Gorelik et al., 2002; Huang et al., 2023). On the other hand, the microbiota can generate protective and functionally important metabolites. The most important example is the conversion of CDCA into UDCA through the action of hydroxysteroid dehydrogenase enzymes (Ridlon et al., 2006; Guzior and Quinn, 2021; Ridlon et al., 2016), which exerts its regulatory effects both through direct actions on receptors and indirectly via changes in BA composition and metabolism (Fiorucci and Distrutti, 2019; Grüner and Mattner, 2021; He et al., 2024).

Beyond FXR and TGR5, BA can interact with additional nuclear and membrane receptors, including the Pregnane X Receptor (PXR), Vitamin D Receptor (VDR), Constitutive Androstane Receptor (CAR), and Sphingosine-1-Phosphate Receptor 2 (S1PR2), contributing to tissue-specific metabolic, inflammatory, and cardiovascular effects. However, FXR and TGR5 remain the primary receptors of interest in the context of cardiometabolic regulation (Wan and Sheng, 2018).

Overall, the systemic biological effects of BA are determined by the composition of the BA pool, receptor engagement profiles, and the balance between hydrophilic and hydrophobic species, with FXR and TGR5 representing the most extensively characterized signaling targets in cardiometabolic contexts. The ratio between hydrophobic and hydrophilic BA, often expressed through the hydrophobicity index, is a key determinant of cytotoxicity, signaling activity, and systemic effects (Li and Apte, 2015; Arab et al., 2017). This balance is largely regulated by the intestinal microbiome; therefore, dysbiosis may shift it toward more hydrophobic and potentially toxic BA, contributing to oxidative stress, chronic inflammation, metabolic disturbances, and increased cardiovascular risk (Tang et al., 2017; Jia et al., 2018; Pushpass et al., 2022; Singh et al., 2019). Conversely, reduced microbial generation of protective metabolites such as UDCA may weaken endogenous cytoprotection. UDCA is distinct from more hydrophobic BA because it can stabilize cellular and mitochondrial membranes, reduce reactive oxygen species (ROS), modulate endoplasmic reticulum (ER) stress, and inhibit apoptotic signalling (Beuers, 2006; Amaral et al., 2009; Rodrigues et al., 1999).

In this context, the microbiota–BA axis represents one of the key regulatory systems in the pathophysiology of metabolic diseases, including type 2 diabetes mellitus (T2DM), where alterations in the microbiome lead to remodelling of the BA profile and functional changes in FXR and TGR5 signaling. Clinically, in individuals with T2DM, an increased proportion of unconjugated secondary BA, particularly DCA, has been associated with a higher cardiovascular risk (Lu et al., 2021; Lu et al., 2024). Beyond T2DM, alterations in the BA profile have been extensively documented after bariatric surgery, including Roux-en-Y gastric bypass (RYGB) and sleeve gastrectomy. Post-bariatric increases in circulating BA- particularly conjugated primary BA and secondary BA- contribute to improved insulin sensitivity, secretion of glucagon-like peptide-1 (GLP-1), and weight-independent metabolic benefits, at least partly through enhanced FXR and TGR5 signaling (Patti et al., 2009; Albaugh et al., 2017). Given that obesity is a major independent cardiovascular risk factor, the BA-mediated component of metabolic improvement after bariatric surgery may carry direct cardiovascular relevance.

Cardiovascular diseases remain the leading cause of morbidity and mortality, and growing evidence suggests that disturbances in FXR/TGR5 signaling, mediated by the microbiome, play a significant role in vascular dysfunction, inflammation, and myocardial injury (Pols et al., 2011; Fiorucci and Distrutti, 2015; Shapiro et al., 2018; Lefebvre et al., 2009; Zhang et al., 2023). In this context, UDCA is of particular interest because it integrates hepatobiliary biology with cardiometabolic and vascular mechanisms.

This review examines the mechanistic basis, experimental evidence, clinical data, and translational limitations of UDCA as a potential cardioprotective agent.

A structured literature search was performed using PubMed, Scopus, and Google Scholar to identify relevant literature published up to 2026. Search terms included keywords related to UDCA, BA signaling, FXR, TGR5, oxidative stress, ROS, mitochondrial dysfunction, ER stress, and cardioprotection. Additional references were identified through manual screening of cited literature. This review represents a narrative synthesis of the available evidence and did not follow a formal systematic review protocol.

## 2 Bile acid signaling and UDCA: a mechanistic positioning

The downstream effects of TGR5 activation differ substantially depending on the cell type. In enteroendocrine L-cells of the intestine, TGR5 activation stimulates adenylyl cyclase, increases intracellular cyclic adenosine monophosphate (cAMP), and activates protein kinase A (PKA), resulting in secretion of GLP-1 and improved glycaemia. In macrophages and Kupffer cells, TGR5 activation reduces nuclear factor kappa B (NF-κB)-mediated production of tumor necrosis factor alpha (TNF-α), interleukin-1 beta (IL-1β), and interleukin-6 (IL-6). In brown adipose tissue, TGR5 signaling activates type 2 iodothyronine deiodinase (D2), promoting thyroid hormone activation and thermogenesis. In the vascular endothelium, TGR5 signaling may modulate endothelial nitric oxide synthase (eNOS) activity and NO production. FXR activation, in contrast, occurs predominantly in hepatocytes and enterocytes. In the liver, FXR suppresses cholesterol 7α-hydroxylase (CYP7A1)-mediated BA synthesis through the Small heterodimer partner (SHP) intermediate. In enterocytes, FXR induces fibroblast growth factor 15/19 (FGF15/19), which acts as an endocrine signal to suppress hepatic BA synthesis via fibroblast growth factor receptor 4 (FGFR4)/beta-Klotho (β-Klotho) signaling. In macrophages, FXR activation reduces inflammatory signaling through NF-κB and NOD-like receptor pyrin domain containing 3 (NLRP3) pathways (Pols et al., 2011; Thomas et al., 2008; Keitel et al., 2007; Inagaki et al., 2005; Kim and Fang, 2018; Guo et al., 2016; Godwin et al., 2000).

UDCA is a hydrophilic BA generated by bacterial conversion of CDCA within the intestinal microbiota. It has been used for decades in hepatobiliary disorders, particularly cholestatic liver disease, where its clinical relevance is based on cytoprotective, anti-apoptotic, and anti-inflammatory properties, as well as a favourable safety profile (Kumar and Tandon, 2001; Trauner et al., 2015; Milivojac et al., 2025b). These established hepatoprotective effects provide the conceptual foundation for examining its potential systemic actions outside the liver.

It is important to clarify the hierarchy of UDCA’s effects on BA pool composition. UDCA primarily exerts its pool-remodeling effects indirectly: by displacing more hydrophobic BA (particularly DCA and LCA) through competition for intestinal absorption and enterohepatic recirculation, and by modifying the gut microbiota composition, thereby shifting microbial BA transformation patterns. This pool remodeling then secondarily influences FXR signaling, because the altered ratio of FXR agonists to antagonists changes the intensity of hepatic FXR activation and the consequent FGF15/19 enterohepatic feedback. UDCA does not act as a strong direct FXR agonist, and its influence on TGR5 signaling is modest and largely indirect. Therefore, the predominant mechanism by which UDCA modulates receptor signaling is through changes in BA pool composition rather than direct receptor activation (Poupon, 2012; Trauner et al., 2015; Fiorucci and Distrutti, 2019).

Quantitative estimates of TGR5 activation suggest that UDCA exhibits substantially lower potency than highly active endogenous ligands such as LCA. Reported EC50 values for UDCA are in the micromolar range, whereas LCA acts in the submicromolar range, supporting the interpretation that UDCA functions as a weak TGR5 ligand. Importantly, these values derive from *in vitro* systems and may not directly reflect *in vivo* receptor engagement due to differences in BA distribution, conjugation, and tissue availability. Where mechanistic evidence specifically derives from tauroursodeoxycholic acid (TUDCA) rather than UDCA, this is noted explicitly in the present review (Lun et al., 2024; Duboc et al., 2014; Pols et al., 2011; Thomas et al., 2008).

UDCA acts as a moderate activator of TGR5, particularly in enteroendocrine and immune cells. Stimulation of TGR5 leads to increased secretion of GLP-1, improved glucose homeostasis, and reduced production of pro-inflammatory cytokines, including TNF-α (Kim and Fang, 2018; Pols et al., 2011). In this way, UDCA contributes to its metabolic and anti-inflammatory effects (Guo et al., 2016; Fiorucci et al., 2018). UDCA may indirectly influence TGR5-related signaling by modulating the BA pool and microbiota-BA interactions, thereby affecting metabolic and inflammatory pathways (Duboc et al., 2014; Wahlström et al., 2016; Poupon, 2012).

UDCA does not function as a strong direct FXR agonist and has repeatedly been shown to lack significant FXR activation in cell-based assays, in contrast to more hydrophobic BA such as CDCA, DCA and LCA (Fujino et al., 2004). However, UDCA can still influence BA signaling indirectly through alterations in BA pool composition, including shifts in the relative abundance of more hydrophobic FXR-active BA and secondary BA metabolites (Song et al., 2015). *In vivo* studies also demonstrate that UDCA participates in BA homeostasis and can modify hepatic BA composition under cholestatic and experimental conditions, although its FXR-mediated transcriptional potency remains limited (Zollner et al., 2006).

These changes in BA composition can modulate FXR-dependent signaling pathways, including regulation of downstream target genes such as SHP, CYP7A1, and bile salt export pump (BSEP), which represent core components of the FXR-SHP feedback axis controlling BA synthesis and transport (Appelman et al., 2021). Importantly, while FXR activation robustly regulates these genes, UDCA itself is not a direct FXR ligand, and its effects on transcriptional output are therefore likely context-dependent and mediated indirectly through alterations in the overall BA milieu rather than receptor binding affinity (Trauner and Boyer, 2003).

In experimental models, BA as a class regulate FXR target gene expression in a dose- and composition-dependent manner, with strong suppression of CYP7A1 and induction of SHP and FGF15/19 signaling observed for more potent FXR agonists, while UDCA exerts comparatively weak or indirect effects on these pathways (Liu et al., 2014). In this context, any UDCA-associated modulation of FXR signaling is likely secondary to its effects on hepatic and intestinal BA composition rather than direct receptor activation.

UDCA has also been shown in animal models to influence BA detoxification and transporter expression, including partial FXR-dependent repression of CYP7A1 and FXR-independent induction of alternative detoxification pathways, suggesting a mixed mechanism of action that combines FXR-dependent and FXR-independent effects (Zollner et al., 2006).

Therefore, the clinical relevance of UDCA lies primarily in its role as a hydrophilic BA that reshapes the BA pool and reduces cytotoxic BA burden, with secondary and indirect modulation of FXR signaling and the enterohepatic FXR-FGF15/19 axis rather than acting as a direct receptor agonist (Song et al., 2015).

**FIGURE 1:**
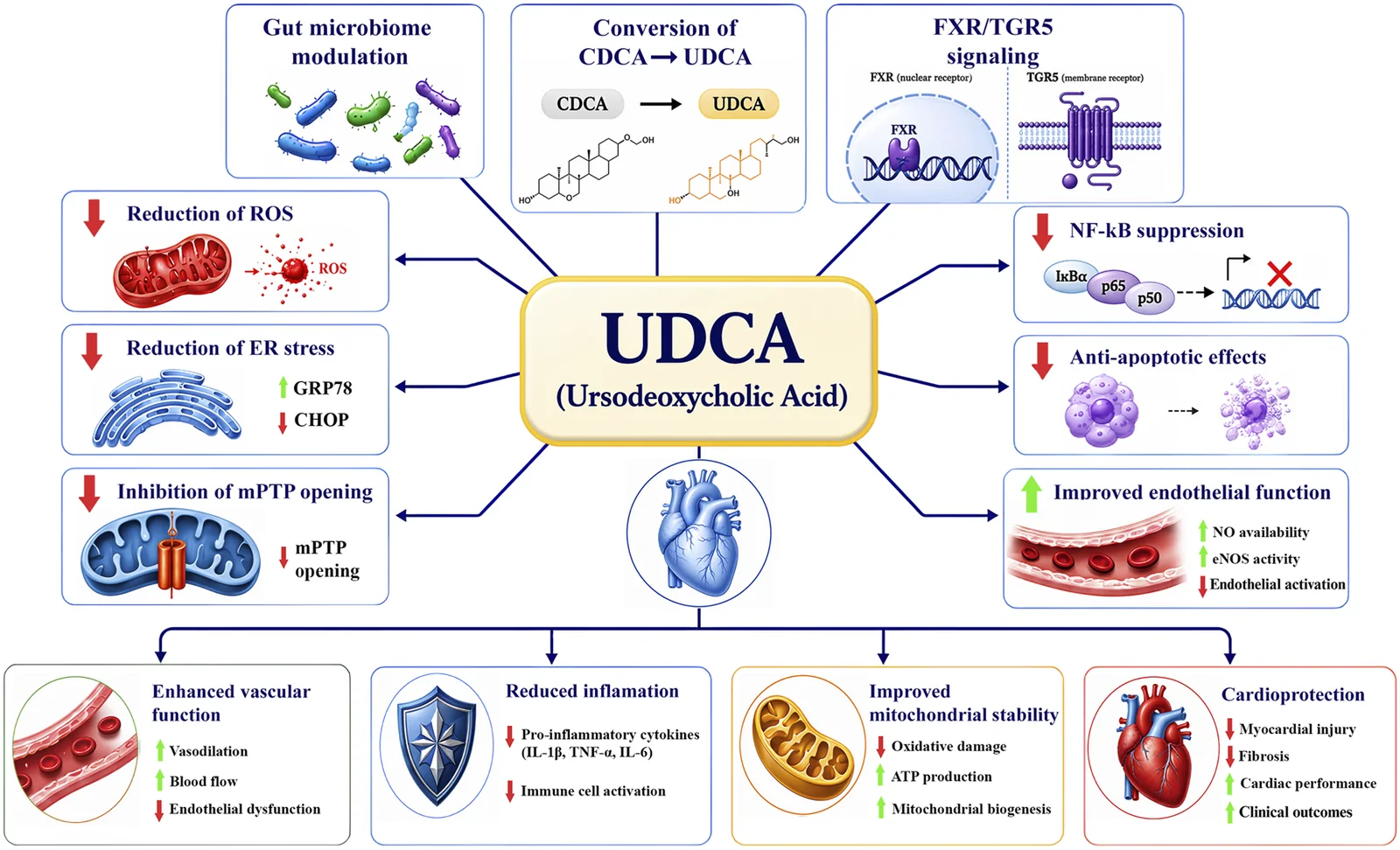
Schematic presentation of UDCA cardioprotective effects. UDCA is formed through microbiome-dependent conversion of CDCA and may exert its effects via remodeling of the BA pool, modulation of FXR/TGR5 signaling pathways, reduction of oxidative stress, stabilization of the endoplasmic reticulum and mitochondria, activation of anti-apoptotic signaling mechanisms, suppression of NF-κB–mediated inflammation, and improvement of endothelial function. These mechanisms provide strong biological plausibility for its potential effects, while direct clinical evidence on cardiovascular outcomes remains limited. Source: Author’s own illustration based on the data presented in Table 1.

Within the framework of the cardiohepatic axis concept, UDCA may contribute to cardiovascular protection through multiple interconnected mechanisms, including modulation of inflammation, stabilization of mitochondria, reduction of ER stress, and improvement of endothelial function (Hakeem et al., 2025; Ma et al., 2017; Rashidbeygi et al., 2025; Li et al., 2025; Fuchs et al., 2025; Skrbić et al., 2026; Lakić et al., 2024). A schematic overview of these mechanisms is presented in Figure 1; proposed pathways and evidence levels are summarized in Table 1.

**TABLE 1:** Proposed cardiovascular protective mechanisms of UDCA.

Target/process	Key pathway(s)	Proposed UDCA effect	Current level of evidence	Cardiovascular relevance
Bile acid pool and microbiome	CDCA-to-UDCA conversion; bile acid pool hydrophobicity (hydrophobic vs. hydrophilic balance) (Ridlon et al., 2006; Guzior and Quinn, 2021; Ridlon et al., 2016; Li and Apte, 2015; Arab et al., 2017)	Shifts the bile acid pool toward a more hydrophilic composition and reduces cytotoxic bile acid exposure	Mechanistic/translational	May reduce systemic metabolic and inflammatory stress
Bile acid receptor signaling	Indirect FXR-FGF15/19 and TGR5-related modulation (Inagaki et al., 2005; Chiang, 2009; Duboc et al., 2014; Wahlström et al., 2016; Poupon, 2012)	Acts mainly through bile acid pool remodeling, not strong direct agonism	Mechanistic	Supports accurate, non-overstated signaling interpretation
Oxidative stress	ROS, lipid peroxidation, Sirt1/Nrf2 (Skrbić et al., 2026; Peng et al., 2019; Gottlieb et al., 1994; Józkowiak et al., 2025)	Reduces ROS and strengthens antioxidant defenses	Experimental + biomarker data	Limits endothelial and myocardial injury
Mitochondrial dysfunction	Membrane potential, mPTP, cytochrome c, ATP (Amaral et al., 2009; Rodrigues et al., 1999; Hausenloy and Yellon, 2007; Youle and Narendra, 2011; Murphy and Steenbergen, 2008; Mihajlović et al., 2024; Del Re et al., 2019)	Stabilizes mitochondria, inhibits mPTP opening, preserves ATP	Strong experimental	Protects cardiomyocytes during ischemic and toxic stress
ER stress	UPR, PERK/eIF2alpha/CHOP, IRE1/XBP1, GRP78 (Förstermann and Sessa, 2012; Han and Kaufman, 2016; Chung et al., 2016; Li et al., 2023; Szegezdi et al., 2006; Wang and Kaufman, 2014)	Reduces maladaptive ER stress and pro-apoptotic UPR activation	Experimental	May attenuate endothelial activation and cardiomyocyte apoptosis
Apoptosis	Bax/Bcl-2, caspase-8/-9/-3, Fas/FasL (Amaral et al., 2009; Whelan et al., 2010; Narula et al., 1999; Yang et al., 2022; Francisco and Del Re, 2023; Mlakar et al., 2015)	Suppresses intrinsic and extrinsic apoptotic signaling	Strong experimental	May limit infarct size, remodeling, and heart failure progression
Inflammation	NF-kB, MAPK, TLR4/MyD88, JAK/STAT, NLRP3, TNF axis (Liu et al., 2017; Hu et al., 2021; Swanson et al., 2019; Libby, 2012; Sparvero et al., 2009; Schultz et al., 2016; von Haehling et al., 2012; Biagioli and Fiorucci, 2021)	Suppresses pro-inflammatory signaling and receptor-mediated TNF activation	Experimental + limited clinical surrogate	May reduce myocardial and vascular inflammatory injury
Endothelial dysfunction	eNOS, NO, KLF2, adhesion molecules, disturbed flow Förstermann and Sessa, 2012; Beuers et al., 1998; Chung et al., 2017; Civelek et al., 2009; Ozcan et al., 2006; Li et al., 2024; Xiong et al., 2025; Nayak et al., 2011; Dabravolski et al., 2022)	Improves vasodilation and reduces endothelial activation	Experimental + small clinical studies	Supports vascular reactivity and microcirculation

A substantial proportion of mechanistic evidence in BA research derives from studies using TUDCA or mixed BA systems; therefore, findings should not be automatically attributed to UDCA. Although UDCA and TUDCA share structural and functional similarities, differences in conjugation, solubility, and tissue distribution may lead to distinct pharmacokinetic and biological profiles (Vang et al., 2014). Accordingly, UDCA-specific interpretations are provided only where directly supported by the original studies.

## 3 UDCA and endothelial function

The evidence for UDCA effects on endothelial function derives predominantly from *in vitro* cell culture models and a small number of clinical studies using surrogate vascular endpoints. No animal model specifically designed to assess endothelial function as a primary cardiovascular outcome has been conducted exclusively with UDCA. Clinical evidence is limited to small randomized trials assessing flow-mediated dilation and peripheral vascular reactivity.

Endothelial dysfunction is an early and central event in cardiovascular disease. It is characterized by reduced NO bioavailability, increased oxidative stress, vascular inflammation, impaired vasodilation, pro-thrombotic activation, and increased expression of adhesion molecules (Förstermann and Sessa, 2012; Chung et al., 2015; Sinisalo et al., 1999; Ma et al., 2004; Nayak et al., 2011; Dabravolski et al., 2022). Because endothelial injury links metabolic stress, hypertension, atherosclerosis, microvascular dysfunction, and heart failure, preservation of endothelial homeostasis is a critical component of any proposed cardioprotective mechanism.

Experimental data suggest that UDCA may improve endothelial phenotype through increased expression of eNOS and the transcription factor Krüppel-like factor 2 (KLF2), both of which contribute to anti-inflammatory and anti-thrombotic endothelial function (Chung et al., 2015; Ma et al., 2004; Nayak et al., 2011).

UDCA has also been reported to reduce oxidative stress and preserve NO signaling, thereby improving vasodilation and microcirculatory function, particularly in the coronary circulation (Beuers et al., 1998; Sinisalo et al., 1999). These effects may translate into improved vascular reactivity and reduced peripheral vascular resistance, although clinical confirmation remains limited.

At the molecular level, UDCA has been shown in endothelial models to increase eNOS phosphorylation at Ser1177 via activation of the phosphoinositide 3-kinase (PI3K)/protein kinase B (Akt) pathway, thereby enhancing NO production and bioavailability (Chung et al., 2015; Vang et al., 2014).

UDCA has also been reported to reduce ER stress markers in vascular and retinal cell models, where ER stress inhibition is associated with improved cellular survival and reduced apoptotic signaling (Vang et al., 2014; Chung et al., 2017). However, direct experimental evidence linking UDCA-induced ER stress reduction to KLF2 induction specifically has not been established; KLF2 is independently suppressed by UPR-associated signaling under disturbed shear stress conditions (Dekker et al., 2002; Parmar et al., 2006), suggesting a plausible but as yet unconfirmed mechanistic link.

In inflammatory endothelial models, UDCA has been shown to reduce intercellular adhesion molecule 1 (ICAM-1) and vascular cell adhesion molecule 1 (VCAM-1) expression, along with inhibition of NF-κB activation and reduction of oxidative stress markers (Chung et al., 2016; Li et al., 2023). These effects are consistent with independently described interactions between NF-κB activation and ROS-dependent inflammatory signaling in endothelial cells, although UDCA-specific integration of NF-κB, ROS, and KLF2 regulatory networks has not been directly demonstrated as a unified pathway in experimental models (Barnes and Karin, 1997; Morgan and Liu, 2011).

In hyperglycemic endothelial models, UDCA has been shown to attenuate receptor for advanced glycation end products (RAGE)-associated inflammatory signaling and reduce endothelial activation markers (Chung et al., 2016). In retinal pericytes, UDCA reduces ER stress-induced apoptosis and improves cell viability under stress conditions (Chung et al., 2017).

One of the most relevant mechanisms involves the inhibition of ER stress in endothelial cells, especially under disturbed flow conditions. Disturbed flow activates the UPR through molecules such as X-box binding protein 1 (XBP1), glucose-regulated protein 78 (GRP78), and C/EBP homologous protein (CHOP), thereby promoting endothelial dysfunction, inflammation, and atherogenesis (Chung et al., 2015; Chung et al., 2017; Civelek et al., 2009; Ozcan et al., 2006; Han and Kaufman, 2016). Chemical chaperone activity—specifically the ability to reduce the accumulation of misfolded proteins within the ER lumen and attenuate UPR activation—has been demonstrated for TUDCA in several key experimental models of vascular and endothelial ER stress (Li et al., 2024; Ozcan et al., 2006). While UDCA shares structural similarity with TUDCA and is presumed to exert analogous chaperone-like properties, direct experimental confirmation of this mechanism specifically for UDCA in endothelial cells remains limited. Accordingly, the anti-atherogenic effects attributed to ER stress attenuation in experimental vascular models should be interpreted as evidence for the bile acid class or for TUDCA specifically, with careful extrapolation to UDCA.

Additional evidence indicates that UDCA and TUDCA may suppress ER-stress-related NF-κB activation, modulate mitogen-activated protein kinase (MAPK), PI3K/Akt, and nuclear factor erythroid 2–related factor 2 (Nrf2) signaling, and reduce the expression of ICAM-1 and VCAM-1 (Chung et al., 2016; Li et al., 2023). The ER-mitochondrial axis further connects these effects, because stabilization of ER function can secondarily improve mitochondrial homeostasis and reduce inflammatory and apoptotic injury in vascular and myocardial cells (Zou et al., 2017; Li et al., 2024; Rizzuto et al., 2012; Vance, 1990).

## 4 UDCA and myocardial protection: oxidative stress, mitochondria, and ER stress

Evidence for UDCA-mediated myocardial protection derives from multiple levels: *in vitro* cardiomyocyte culture experiments, isolated perfused heart (Langendorff) preparations, rodent models of ischemia-reperfusion injury, isoproterenol-induced cardiac injury models, and a limited number of post-infarction remodeling studies. No dedicated prospective randomized clinical trial has examined hard myocardial outcomes as a primary endpoint for UDCA. Some evidence in this section derives from TUDCA studies, which is explicitly noted.

Oxidative stress is a central mechanism of myocardial injury and results from increased ROS generation combined with impaired endogenous antioxidant defence. Elevated ROS levels damage lipids, proteins, and DNA, impair cardiomyocyte structure and function, and reduce NO bioavailability, thereby linking myocardial injury with endothelial dysfunction and vascular tone abnormalities (Xiong et al., 2025; Wang et al., 2026; Çelik et al., 2025; Józkowiak et al., 2025). Major intracellular sources of ROS in the cardiovascular system include nicotinamide adenine dinucleotide phosphate (NADPH) oxidases and mitochondria (Bedard and Krause, 2007; Konior et al., 2014). Their activation can amplify MAPK signaling, particularly p38 and c-Jun N-terminal kinase (JNK), and stimulate NF-κB-dependent inflammatory responses (Kibel et al., 2020; Peng et al., 2019; Wróbel-Nowicka et al., 2024; Brown and Griendling, 2015; Rose et al., 2010).

Mitochondrial dysfunction is a fundamental driver of cardiomyocyte injury in ischemia-reperfusion injury, heart failure, and cardiometabolic disease. It is characterized by impaired adenosine triphosphate (ATP) production, increased ROS generation, loss of mitochondrial membrane potential, and disruption of mitochondrial membrane integrity (Zhou and Tian, 2018; Zhang et al., 2025). A decisive event is the opening of the mitochondrial permeability transition pore (mPTP), which triggers loss of membrane potential, mitochondrial swelling, and activation of necrotic or apoptotic cell death (Bonora and Pinton, 2014; Halestrap and Richardson, 2015). Pathways such as PI3K/Akt and reperfusion injury salvage kinase (RISK) can protect cardiomyocytes by inhibiting mPTP opening, partly through Akt-mediated inhibition of glycogen synthase kinase 3 beta (GSK-3β), while PTEN-induced putative kinase 1 (PINK1)/Parkin-mediated mitophagy contributes to mitochondrial quality control (Hausenloy and Yellon, 2007; Ong and Hausenloy, 2010; Halestrap and Richardson, 2015; Youle and Narendra, 2011; Murphy and Steenbergen, 2008).

ER stress is another major mechanism of myocardial injury, particularly in ischemia, reperfusion, diabetes, obesity, and lipotoxic states (Wang et al., 2018; Ren et al., 2021). The UPR initially attempts to restore ER homeostasis through signalling by protein kinase RNA-like endoplasmic reticulum kinase (PERK), inositol-requiring enzyme 1 (IRE1), and activating transcription factor 6 (ATF6). However, sustained or severe ER stress induces pro-apoptotic signaling, including CHOP activation, caspase-related pathways, and mitochondrial crosstalk (Ren et al., 2021; Hotamisligil, 2010; Toko et al., 2010; Szegezdi et al., 2006; Wang and Kaufman, 2014; Glembotski, 2007). This ER-mitochondrial interaction is particularly important in cardiomyocytes, where metabolic demand is high and cellular survival depends on coordinated protein folding, calcium handling, and energy generation.

UDCA and its conjugate TUDCA may provide cardioprotection by simultaneous targeting of oxidative stress, mitochondrial dysfunction, and ER stress (Reilly-O’Donnell et al., 2025; Amaral et al., 2009). The evidence from Zhang and Wang specifically derives from TUDCA administration in neonatal rat cardiomyocytes (Zhang and Wang, 2018).

Experimental studies have shown that UDCA reduces ROS production, stabilizes mitochondrial membrane potential, preserves redox balance, inhibits mPTP opening, prevents cytochrome c release, and attenuates caspase activation (Chung et al., 2016; Perez and Briz, 2009; Reilly-O'Donnell et al., 2025; Amaral et al., 2009; Rodrigues et al., 1998a). It also stabilizes mitochondrial membranes by reducing Bcl-2–associated X protein (Bax) translocation (Rodrigues et al., 1999). In models of ischemia-reperfusion injury and catecholamine-induced stress, UDCA has been associated with reduced oxidative damage, preserved ATP synthesis, improved cardiomyocyte energy stability, lower caspase-3 activation, and more favourable regulation of the B-cell lymphoma 2 protein (Bcl-2)/Bax ratio (Reilly-O'Donnell et al., 2025; Ferraro et al., 2020; Lee et al., 1999).

These effects may involve activation of pro-survival signaling pathways, including PI3K/Akt and extracellular signal-regulated kinase (ERK), as well as enhancement of endogenous antioxidant defences through Sirtuin 1 (Sirt1)/Nrf2 signaling (Mihajlović et al., 2024; Skrbić et al., 2026; Rashidbeygi et al., 2025; Song et al., 2021). The antioxidant effects of UDCA operate through multiple complementary mechanisms. UDCA activates Sirt1, a NAD + -dependent deacetylase that promotes Nrf2 nuclear translocation and transcription of downstream antioxidant genes including heme oxygenase-1 (HO-1), NAD(P)H quinone dehydrogenase 1 (NQO1), superoxide dismutase (SOD), and glutathione peroxidase (GPx). This Sirt1/Nrf2 axis represents a master antioxidant regulatory module that coordinates the cellular response to oxidative injury (Skrbić et al., 2026; Mihajlović et al., 2024; Rodrigues et al., 1998b). Separately, UDCA reduces mitochondrial ROS generation by stabilizing the inner mitochondrial membrane potential and preventing electron leak at complexes I and III. Inhibition of mPTP opening by UDCA further prevents the release of pro-oxidant mitochondrial contents into the cytoplasm (Rodrigues et al., 1999; Amaral et al., 2009).

Regarding ER stress, UDCA acts primarily as a chemical chaperone—it reduces the burden of misfolded proteins within the ER lumen, attenuating activation of the three canonical UPR sensors: PERK/eIF2α/CHOP, IRE1/XBP1, and ATF6. The net result is reduced CHOP-mediated transcription of pro-apoptotic genes, preserved ER calcium homeostasis, and attenuation of the ER-mitochondria apoptotic axis (Ozcan et al., 2006; Szegezdi et al., 2006; Wang and Kaufman, 2014; Han and Kaufman, 2016).

In experimental models, including Langendorff-perfused hearts, BA derivatives have been shown to improve contractile function and stabilize myocardial electrical activity (Ferraro et al., 2020; Reilly-O'Donnell et al., 2025). In chronic post-infarction remodelling processes, UDCA reduced fibrosis and preserved left ventricular systolic function, supporting its potential relevance beyond acute cellular injury (Reilly-O'Donnell et al., 2025).

## 5 Anti-apoptotic effects of UDCA in the cardiovascular system

Anti-apoptotic evidence for UDCA in the cardiovascular context is primarily experimental, derived from *in vitro* studies and animal models. Human data directly assessing apoptotic markers in cardiac tissue following UDCA administration are not available. Several key mechanistic studies cited in this section used TUDCA rather than UDCA, and are explicitly identified as such.

Apoptosis contributes to the progressive loss of cardiomyocytes in ischemia-reperfusion injury, myocardial infarction, and heart failure. Although apoptosis is a regulated form of cell death without the same degree of immediate inflammatory disruption as necrosis, excessive activation reduces the number of functional cardiomyocytes and accelerates the deterioration of myocardial performance (Del Re et al., 2019; Dong et al., 2019).

Cardiomyocyte apoptosis occurs through intrinsic mitochondrial and extrinsic receptor-mediated pathways. The intrinsic pathway involves an altered Bax/Bcl-2 balance, mitochondrial outer membrane permeabilization, cytochrome c release, and activation of caspase-9 and caspase-3. The extrinsic pathway involves death receptor signaling, including First apoptosis signal receptor/Fas ligand (Fas/FasL) and caspase-8 activation, with convergence on executioner caspase-3 (Zhang and Wang, 2018; Whelan et al., 2010; Narula et al., 1999; Bock and Tait, 2020; Yang et al., 2022; Bertheloot et al., 2021; Zhang et al., 2019). It is important to note that some mechanistic evidence in cardiomyocytes, including that reported by Zhang and Wang (2018), was obtained using TUDCA, and may not fully reflect UDCA-specific effects.

UDCA exerts anti-apoptotic effects by modulating both pathways. Experimental data show that UDCA inhibits caspase activation, particularly caspase-8 and caspase-9, stabilizes mitochondrial membranes, and shifts Bax/Bcl-2 regulation toward cell survival (Amaral et al., 2009; Rodrigues et al., 1998a). In ischemia-reperfusion models, UDCA reduced cardiomyocyte apoptosis and infarct size, with activation of PI3K/Akt signaling and preservation of mitochondrial function (Rajesh et al., 2005). In isoproterenol-induced myocardial injury, UDCA reduces oxidative stress and promotes an anti-apoptotic shift in the Bcl-2/Bax ratio (Mihajlović et al., 2024).

In models of BA-induced and hypoxia-related injury, including studies where TUDCA was used as the experimental agent, BA derivatives have been shown to reduce apoptotic markers, lower ROS levels, and preserve myocardial ultrastructure (Kalogeris et al., 2012). Long-term post-infarction remodeling studies further indicate that UDCA may reduce cardiomyocyte loss and fibrosis, thereby contributing to the preservation of cardiac function (Reilly-O'Donnell et al., 2025; Lagranha et al., 2010; Kalogeris et al., 2012; Rodrigues et al., 1998a; Ferraro et al., 2020).

In summary, suppression of apoptotic signaling represents one of the most consistent mechanistic features of UDCA cardioprotection in experimental models, with the caveat that a portion of the supporting evidence derives specifically from studies using TUDCA rather than UDCA.

## 6 Anti-inflammatory and immunomodulatory effects

Evidence for UDCA anti-inflammatory effects in the cardiovascular system derives mainly from *in vitro* macrophage and endothelial cell experiments, with supportive data from animal models of endotoxin-induced and ischemic myocardial injury. Human clinical data on UDCA effects on systemic inflammatory cytokines are inconsistent and largely based on small studies; no large-scale clinical trial has confirmed meaningful reduction of inflammatory biomarkers as a primary cardiovascular endpoint.

Inflammation plays a central role in the initiation and progression of myocardial and vascular injury, particularly during ischemia, reperfusion, atherosclerosis, metabolic disease, and heart failure (Sagris et al., 2024; Francisco and Del Re, 2023). Inflammatory activation increases the production of pro-inflammatory cytokines such as TNF-α, IL-6, and IL-1β, promotes immune cell recruitment, and amplifies both local and systemic inflammatory injury (Mlakar et al., 2015). Chronic inflammation also increases oxidative stress and cell death, creating a feed-forward cycle of tissue damage.

At the molecular level, myocardial inflammation is regulated by several interconnected pathways. NF-κB is a central transcription factor that drives inflammatory gene expression (Matsumori, 2023; Liu et al., 2017; Vinten-Johansen et al., 2007; Saini et al., 2005; Frangogiannis, 2015). Innate immune signaling through Toll-like receptor 4 (TLR4)/Myeloid differentiation primary response (MyD88) contributes to the initiation of inflammatory responses (Yang et al., 2016; Su et al., 2018) while the Janus kinase (JAK)/Signal Transducer and Activator of Transcription (STAT) pathway transduces cytokine signalling (Hu et al., 2021; O'Shea et al., 2015). Activation of the NLR family pyrin domain containing 3 (NLRP3) inflammasome promotes maturation and release of IL-1β, further amplifying inflammation and tissue injury (Swanson et al., 2019). The final consequence is progressive loss of functional cardiomyocytes, extracellular matrix remodeling, fibrosis, and worsening cardiac function (Wróbel-Nowicka et al., 2024; Mlakar et al., 2015; Frangogiannis, 2014).

UDCA has been reported to exert anti-inflammatory and immunomodulatory effects that may contribute to cardiovascular protection. Experimental studies suggest that UDCA-related compounds may modulate NF-κB signaling, MAPK cascades, and RAGE-associated inflammatory pathways in macrophages and endothelial cells. However, several of these mechanistic observations have been demonstrated in studies using TUDCA or broader BA–based experimental systems, and therefore may not be exclusively attributable to UDCA (Libby, 2012; Schmidt et al., 2000; Sparvero et al., 2009; Hotamisligil, 2010; Ko et al., 2017; Milivojac et al., 2025a).

These effects are relevant because the same inflammatory pathways participate in atherosclerosis, diabetic vascular injury, ischemia-reperfusion injury, and heart failure progression.

UDCA and related BA derivatives demonstrate anti-inflammatory and cardioprotective effects across experimental cardiac models, although the relative contribution of UDCA-specific versus derivative-mediated mechanisms (including TUDCA-based effects) remains incompletely defined (Reilly-O'Donnell et al., 2025; Ferraro et al., 2020; Schultz et al., 2016).

## 7 Clinical evidence and systemic effects

Clinical evidence for the cardioprotective effects of UDCA remains limited. Available studies suggest potential beneficial effects on cardiovascular and inflammatory parameters, including vascular function and circulating inflammatory markers. In this context, some reports and mechanistic interpretations of anti-inflammatory signaling raise the possibility that UDCA may influence pathways related to TNF signaling, including soluble TNF receptor 1 (sTNFR1), although this has not been consistently or directly demonstrated across clinical studies (Çelik et al., 2025; von Haehling et al., 2012). Effects on endothelial function include improvement in both NO-dependent and NO-independent vasodilation (Sinisalo et al., 1999; Ma et al., 2004).

One of the most relevant clinical observations comes from a randomized, double-blind crossover study in patients with chronic heart failure, in which UDCA improved peripheral blood flow, post-ischemic perfusion, and vascular reactivity. These findings suggest potential vascular benefits of UDCA, although its effects on classical systemic inflammatory cytokines appear limited, indicating that its actions may be more closely related to vascular and endothelial modulation rather than broad immunosuppression (von Haehling et al., 2012).

Similar findings have been reported in patients with coronary artery disease, where UDCA improved endothelium-dependent vasodilation and microvascular reactivity, including NO-independent regulation of vascular tone (Sinisalo et al., 1999). Such effects are important because endothelial dysfunction is a central pathophysiological event in atherosclerosis, coronary microvascular disease, and heart failure.

Meta-analyses of randomized controlled trials suggest that UDCA may improve selected cardiometabolic risk factors and oxidative stress indices, including total antioxidant capacity and lipid peroxidation markers (Rashidbeygi et al., 2025). However, effects on conventional inflammatory cytokines have not been consistently confirmed. These findings support the view that UDCA may act through a complex interaction between BA signaling, endothelial biology, oxidative stress regulation, and the gut-liver-heart axis rather than through a single dominant anti-inflammatory mechanism.

Additional clinical evidence comes from patients with T2DM, where UDCA has been associated with improved oxidative status and increased antioxidant enzyme activity, including SOD and glutathione-related defences (Badnjević-Čengić et al., 2026; Lakić et al., 2024). Although these studies did not primarily assess cardiovascular outcomes, their results have translational relevance because oxidative stress, insulin resistance, endothelial dysfunction, and cardiometabolic risk are tightly connected. In selected populations, such as pregnant women with gestational diabetes, UDCA has demonstrated biological activity on cardiometabolic parameters, but without clear effects on global cardiac function (Matallana et al., 2025).

Overall, clinical evidence remains limited and primarily based on surrogate endpoints as shown in Table 2. In summary, the available human evidence can be stratified as follows: (1) mechanistic/*in vitro*: robust; (2) animal model: strong and consistent; (3) human surrogate-marker studies: limited, with small sample sizes and short follow-up; (4) clinical outcome evidence: absent. No study has demonstrated reduction in myocardial infarction, stroke, heart failure hospitalization, or cardiovascular mortality attributable to UDCA.

**TABLE 2:** Summary of human studies examining UDCA in cardiovascular or cardiometabolic contexts.

Study	Design	Population (n)	UDCA dose	Duration	Endpoints	Main findings	Limitations
Sinisalo et al. (1999)	Randomized, double-blind, placebo-controlled crossover trial	Patients with coronary heart disease and endothelial dysfunction (n = 11)	13–19 mg/kg/day	6 weeks	Endothelial-dependent and endothelial-independent forearm vasodilation	Improved endothelium-dependent, NO-independent vasodilation; enhanced vascular responses during impaired NO signaling	Small sample size, short duration, surrogate vascular endpoints only, no clinical outcomes
von Haehling et al. (2012)	Randomized, double-blind, placebo-controlled crossover trial	Patients with chronic heart failure (n = 17)	500 mg/day	4 weeks per phase	Peripheral blood flow, post-ischemic vascular reactivity, endothelial function, inflammatory markers	Improved post-ischemic peripheral perfusion and vascular responsiveness; no effect on exercise capacity or inflammatory cytokines	Small sample size, short duration, surrogate endpoints only, no hard cardiovascular outcomes
Lakic et al. (2024)	Randomized, double-blind, placebo-controlled trial	Patients with T2DM (n = 60)	1500 mg/day	8 weeks	Oxidative stress markers, antioxidant enzymes, metabolic and anthropometric parameters, blood pressure, liver enzymes, inflammatory markers	Increased antioxidant enzyme activity and improved redox status; improved body mass index (BMI), metabolic and liver parameters	Single-center study, small sample size, short duration, surrogate biomarkers only, no cardiovascular outcomes
Matallana et al. (2025) (GUARDS trial)	Randomized, double-blind, placebo-controlled clinical trial	Women with gestational DM (GDM) (n = 113)	500 mg twice daily (1 g/day)	10–14 weeks (24–28 weeks gestation to delivery)	Glycemic control (primary), OGTT, maternal metabolic outcomes, fetal cardiac function, neonatal outcomes	No significant reduction in fasting glucose or primary glycemic endpoint; no meaningful differences in secondary outcomes	Short-term follow-up, negative primary endpoint, surrogate outcomes, no cardiovascular outcomes, specific population
Rashidbeygi et al. (2025)	Systematic review and meta-analysis of randomized controlled trials	Mixed cardiometabolic populations (∼1796 participants)	Heterogeneous across studies	Heterogeneous	Body mass index (BMI), blood pressure, inflammatory markers	Small reduction in BMI and diastolic blood pressure (DBP); increase in systolic blood pressure (SBP); no effect on weight loss or inflammatory markers Oxidative stress markers, antioxidant capacity	Heterogeneity among studies; surrogate biomarkers; no clinical cardiovascular outcomes, variability in included trials

## 8 Limitations of current evidence

Despite growing experimental and translational interest in UDCA as a potential cardioprotective agent, several important limitations should be considered when interpreting the available evidence.

First, a substantial proportion of current knowledge derives from *in vitro* experiments and animal models. Although these studies provide important mechanistic insights, they do not fully replicate the complexity of human cardiovascular disease. Moreover, species-specific differences in BA composition, receptor expression, and metabolic regulation may limit direct translation of experimental findings to clinical settings. Second, some experimental studies employ UDCA concentrations or dosing regimens that may not directly reflect clinically achievable exposure, raising questions regarding dose relevance and translational applicability. Third, the available clinical evidence remains limited. Most studies involve relatively small sample sizes, short treatment durations, and proof-of-concept designs. Consequently, the majority of human data are based on surrogate endpoints such as endothelial function, vascular reactivity, inflammatory biomarkers, oxidative stress parameters, or metabolic indices rather than hard cardiovascular outcomes. Fourth, uncertainty remains regarding the patient populations most likely to benefit from UDCA therapy. Existing studies have examined heterogeneous populations, including patients with heart failure, coronary artery disease, diabetes, metabolic disorders, and hepatobiliary disease, making it difficult to identify specific cardiovascular indications for treatment. Fifth, publication bias cannot be excluded, as positive mechanistic and preclinical findings may be more likely to be reported than neutral or negative results.

Finally, the cardiovascular effects of UDCA should be interpreted within the context of contemporary guideline-directed therapies for cardiovascular disease, for which robust outcome data are available. At present, evidence supporting UDCA remains largely mechanistic and hypothesis-generating rather than outcome-driven.

## 9 Conclusion

UDCA is a biologically plausible cardioprotective candidate with convergent experimental evidence supporting its effects on oxidative stress, mitochondrial integrity, ER stress, apoptosis, inflammation, and endothelial function. However, the transition from experimental promise to clinical utility requires rigorous validation.

Future translational research should focus on identifying specific target populations most likely to benefit from UDCA, including patients with high cardiovascular risk phenotypes such as metabolic syndrome, diabetes, and atherosclerotic cardiovascular disease. Clinical trials should incorporate well-defined and clinically meaningful endpoints, including major adverse cardiovascular events, hospitalization rates, and relevant mechanistic biomarkers. Safety monitoring should systematically assess hepatic function, gastrointestinal tolerance, and long-term metabolic effects. Additionally, dosing strategies remain to be standardized, warranting dedicated dose-finding studies prior to large-scale clinical trials. Ultimately, well-designed, placebo-controlled phase II studies followed by multicenter, randomized, long-term phase III trials are required to establish the clinical utility of UDCA in cardiovascular disease.
